# Seasonal and Sex-Specific Liver Plasticity in Brown Trout: Estrogen-Responsive Targets and Cell Turnover Dynamics

**DOI:** 10.3390/ani16071073

**Published:** 2026-04-01

**Authors:** Amândio de Barros, Diana Santos, Tiago Lourenço, Célia Lopes, Tânia Vieira Madureira, Eduardo Rocha

**Affiliations:** 1 Group of Animal Morphology and Toxicology, Interdisciplinary Centre of Marine and Environmental Research (CIIMAR/CIMAR), University of Porto, Terminal de Cruzeiros do Porto de Leixões, Av. General Norton de Matos s/n, 4450-208 Matosinhos, Portugal; up201907524@edu.icbas.up.pt (A.d.B.); up201707353@edu.icbas.up.pt (T.L.); cclopes@icbas.up.pt (C.L.); 2 Laboratory of Histology and Embryology, Department of Microscopy, ICBAS—School of Medicine and Biomedical Sciences, University of Porto, Rua Jorge Viterbo Ferreira 228, 4050-313 Porto, Portugal; dianasantiagosantos@gmail.com

**Keywords:** oestrogen-responsive pathways, hepatocyte remodelling, proliferation and apoptosis markers, teleost reproductive physiology, vitellogenin and zona pellucida

## Abstract

The brown trout (*Salmo trutta*) is a commercially and ecologically significant salmonid fish, yet its hepatic cellular and functional dynamics throughout the reproductive cycle remain poorly characterised, particularly in males. This study investigated seasonal and sex-specific liver plasticity across four reproductive stages: spawning capable (December), regressing (March), regenerating (July), and developing (November). We quantified mRNA and protein abundance of key oestrogen-responsive targets—vitellogenin (VtgA) and zona pellucida (ZP) proteins—alongside cell turnover markers, caspase 3 (Casp3) and proliferating cell nuclear antigen (PCNA). These molecular endpoints were integrated with stereological analyses to estimate hepatocyte, nuclear, and cytoplasmic volumes. Results revealed stage-dependent mobilisation and transient hepatic retention of reproductive proteins; females exhibited stronger vitellogenic signatures and more pronounced seasonal shifts than males. Although *VtgA* and *ZP* mRNA levels peaked during the developing and spawning-capable stages, males maintained low but consistent levels throughout the cycle, indicating constitutive hepatic oestrogen sensitivity. Regarding cell turnover, PCNA protein data indicated heightened proliferative activity during the spawning-capable and regressing stages. In contrast, while *Casp3* mRNA levels remained stable across all stages, protein detection suggested a post-transcriptional increase in apoptotic signalling during the developing phase, consistent with controlled tissue remodelling rather than extensive cell loss. Stereological data confirmed enlarged hepatocyte and nuclear volumes during periods of high secretory and proliferative demand. Overall, these findings demonstrate significant stage-dependent and sex-specific plasticity in brown trout liver, providing a robust reference framework for ecological monitoring, endocrine disruption assessments, and studies of teleost reproductive physiology.

## Simple Summary

Brown trout is a vital species to many riverine ecosystems, is important in recreational angling and aquaculture, and serves as a key indicator of environmental health. While research typically focuses on reproductive organs, we investigated how the liver—the body’s metabolic engine—structurally and functionally adapts throughout the year in both sexes. In this study, we followed adult brown trout through four reproductive phases, integrating gene expression, protein detection, and stereology with changes in the hepatosomatic index. We examined products essential for egg development, markers of cell renewal, and changes in cell and nuclear volume. Our findings reveal that female livers undergo profound seasonal transformations, but surprisingly, male livers also produce low but consistent amounts of these “female-specific” molecules. This reveals a fundamental, and previously underappreciated, sensitivity to sex hormones in males. We also observed how liver cells physically adapt to meet the demands of the reproductive cycle. Understanding this natural plasticity is essential for the sustainable management of wild and farmed trout, namely by sustaining the correct distinction between adaptive and pathological changes. Moreover, it provides a vital baseline for using liver changes as early warning signs of pollution and hormone-disrupting chemicals, helping to safeguard freshwater ecosystems for society.

## 1. Introduction

Brown trout (*Salmo trutta*) is a Salmonidae fish species that has been successfully introduced into more than 24 countries worldwide, reflecting its high ecological plasticity [1]. Owing to its economic and ecological relevance, brown trout plays a relevant role in aquaculture, particularly for recreational fisheries and restocking [2]. It is also regularly used as a scientific model organism, namely in environmental monitoring (e.g., [3,4,5]) and physiological studies (e.g., [6]).

Brown trout sexual maturity typically occurs between two and three years of age, although this timing varies with life-history strategy [4]. As in other salmonids, its reproduction follows a well-defined annual cycle, during which physiological changes are tightly regulated by environmental cues, such as photoperiod and temperature [7]. Although gonadal adaptability throughout the cycle has been well described [8,9,10], other organs that play key roles in reproductive regulation have received comparatively little attention. The liver is particularly relevant to reproduction because of its central role in oestrogen-mediated processes essential for gonadal maturation, particularly oocyte development. Under estrogenic stimulation, the liver synthesises vitellogenins (Vtg), the major yolk precursors, as well as zona pellucida (ZP) proteins, which contribute to the formation of the egg envelope [11,12]. Specifically, during vitellogenesis, 17β-estradiol (E2) binds to hepatic oestrogen receptors, activating oestrogen-response elements in *VTG* and *ZP* genes and promoting their transcription [11,12]. Despite this, multiple lines of evidence have shown that these pathways are not exclusively oestrogen-driven. Studies in salmonid hepatocytes under androgen modulation showed upregulation of *VtgA* and *ZP* mRNA levels following exposure to testosterone and dihydrotestosterone [13,14]. Furthermore, basal expression of Vtg- and ZP-related genes and proteins has been reported in males across several teleost species, with detection observed in plasma and diverse solid tissues [10,15,16,17,18].

Across the breeding cycle, additional indicators of shifts in liver activity have been reported. During vitellogenesis, an increase in the volume density of the rough endoplasmic reticulum (RER) and Golgi apparatus per hepatocyte was described, reflecting enhanced secretory activity of hepatocytes [19]. These changes, together with seasonal variations in the hepatosomatic index (HSI) and hepatocyte number [20], suggested periods of programmed cell proliferation and cell death. Supporting this hypothesis, liver transcriptomic analyses of male brown trout exposed to E2 showed upregulation of cell-proliferation-related pathways, together with a downregulation of apoptosis-related genes [21]. In contrast, the data about the effects of androgens on hepatic cell turnover in salmonids remain limited, as most of the few studies have focused on metabolic and peroxisomal pathways rather than on proliferation and programmed cell death markers [13,14]. These knowledge gaps warrant investigation into liver dynamics in critical periods of the brown trout reproductive cycle, particularly in males.

This study aims to characterise selected seasonal and sex-specific changes in hepatic oestrogen-responsive targets and cell turnover in adult male and female brown trout. Vtg and ZP proteins and gene expression were assessed together with markers of apoptosis (caspase 3—Casp3) and cell proliferation (proliferating cell nuclear antigen—PCNA), across four key phases of the reproductive cycle: spawning capable (December), regressing (March), regenerating (July), and developing (November) [10]. Liver samples from females and males were analysed by immunohistochemistry and molecular analysis. Stereology was also used to estimate the hepatocyte volume, nuclear and cytoplasmic volumes, and the nucleus-to-cell volume ratio. This integrated approach provides new insights into liver function and regulation across distinct brown trout reproductive stages, with particular emphasis on hepatic changes in males, a largely understudied sex.

## 2. Materials and Methods

### 2.1. Animals

Brown trout (3–4 years old) were obtained from Torno Aquaculture Station (Amarante, Portugal). The fish were hatched and reared on-site in a continuous flow-through system supplied by a pristine natural mountain stream (Ribeiro do Ramalhoso), reflecting the river’s ambient water parameters and photoperiod. Fish were fed a commercial trout diet (ICNF7MM, AQUASOJA, São João Ovar, Portugal) daily, fasting only the day before sampling.

Six males and six females from the same age cohort were sampled at each of the four reproductive stages, giving a total of 24 individuals per sex. Sampling took place in December 2017 (spawning capable), March 2018 (regressing), July 2018 (regenerating), and November 2018 (developing). The sampling was performed on-site, and animal handling complied with the Portuguese Decree No. 113/2013, which transposes the EU Directive No. 2010/63 on animal protection for scientific purposes. The animals analysed in this study were the same individuals as those used in a previous study [10].

### 2.2. Sampling Procedures

Fish were euthanised individually by an overdose of ethylene glycol monophenyl ether (2 to 3 mL/L) (Merck, Darmstadt, Germany), and, after necropsy, small liver fragments (≈1 cm^3^) were systematically sampled, fixed in 10% buffered formalin for 48 h, at room temperature, and subsequently used for immunohistochemistry procedures. Additional liver samples were also collected, snap-frozen in liquid nitrogen, and stored at −80 °C for RNA extraction. Body, liver, and gonad biometric parameters were calculated as described previously [10]. For the present study, an HSI was calculated as a percentage of liver-to-body mass, excluding the gonadal mass.

### 2.3. Histological Procedure

Three randomly selected liver fragments of each fish were fixed and processed using a standard histological protocol (ethanol 70%, 90%, 96%, 99.9%, ethanol 99.9%/xylene, xylene, and paraffin) in an automatic tissue processor (Leica TP 1020, Wetzlar, Germany). Fragments were then embedded in paraffin using an embedding centre (Leica EG 1140 C, Germany). From each of the three fragments, four glass slides were obtained, each containing three consecutive serial sections (4 µm thick), produced using an automatic rotary microtome (Leica RM2255, Germany). The slides were subsequently processed for immunohistochemistry, with one slide allocated per antibody.

### 2.4. Immunohistochemistry

Immunohistochemical analyses were performed using antibodies against Vtg, ZP, Casp3, and PCNA. The Vtg antibody (PO-1, Biosense Laboratories AS, Bergen, Norway, REF: V01409201-100) was a rabbit polyclonal anti-Arctic char antibody (PO-1), used at a dilution of 1:2500. The ZP antibody (ZRP-12, Biosense Laboratories AS, Bergen, Norway, REF: Z03402202-100) was a rabbit anti-salmon polyclonal antibody (O-146), diluted 1:3000. The Casp3 antibody (Abcam, Cambridge, UK, REF: AB4051) was a rabbit anti-human polyclonal antibody, used at a dilution of 1:400. PCNA antibody (Santa Cruz Biotechnology, Dallas, TX, USA, REF: SC-56) was a mouse monoclonal IgG2a k antibody, diluted 1:3000. Dilutions were prepared in PBS supplemented with 5% bovine serum albumin (BSA). Despite not being trout-specific, these antibodies have demonstrated cross-reactivity and consistent immunostaining in other fish species [13,22,23]. Additionally, PCNA is a highly conserved protein across species [24,25], a characteristic shared by Casp3 [26,27]. Finally, the Vtg antibody is guaranteed by the manufacturer to cross-react with brown trout, while the ZP antibody is validated for a variety of species, including salmonids.

The slides were deparaffinised and then rehydrated through a graded ethanol series of decreasing concentrations. Antigen retrieval was subsequently performed. For Vtg and PCNA, antigen retrieval was carried out using Tris-EDTA buffer (10 mM Tris base, 1 mM EDTA, 0.05% Tween 20, pH 9.0), with slides heated in a microwave oven at 500 W for 15 min. For ZP and Casp3, citrate buffer (0.01 M, pH 6.0) was used, and the slides were kept in the pressure cooker for 3 min after the maximum pressure was reached. The slides were then cooled at room temperature, and endogenous peroxidase activity was blocked by incubating the sections for 10 min in a 3% hydrogen peroxide solution (Merck, Germany, REF: 1.0710.1000) prepared in methanol (VWR Chemicals, Fontenay-sous-Bois, France, REF: 20903.415). The immunolabelling protocol was performed using the NovoLink™ Polymer Detection System (Leica Biosystems, Wetzlar, Germany), according to the manufacturer’s instructions. Primary antibody incubation was performed in a humidified chamber for 2 h.

Within each slide, one section served as a negative control, in which the primary antibody was replaced with PBS containing 5% BSA. Positive control sections of adult female brown trout liver (for Vtg and ZP) or digestive tract (for Casp3 and PCNA) were also included in every immunohistochemistry batch. Immunoreactivity was visualised using 3,3′-diaminobenzidine (DAB) (working solution prepared from reagents supplied in the NovoLink™ kit), and sections were counterstained with Mayer’s hematoxylin (Merck) for 1 min. Slides were then dehydrated and mounted with Q Path^®^ Coverquick 2000 (VWR Chemicals).

### 2.5. Immunostaining Analyses

#### 2.5.1. Micrograph Acquisition

For each Vtg-, ZP-, and Casp3-immunostained slide, one tissue section was randomly selected and divided into four quadrants. One quadrant was randomly excluded, and two micrographs of randomly selected hepatic parenchyma areas were taken from each of the remaining quadrants, resulting in 6 images per slide and a total of 18 images per fish (derived from three slides per fish). Digital images were captured in bright-field mode with a camera (EP50, Olympus, Tokyo, Japan) and an optical microscope (CX21, Olympus, Japan), using a 100× objective lens. All images were used for semi-quantitative analyses.

#### 2.5.2. Semi-Quantitative Analyses of Vtg, ZP, and Casp3 Immunolabelling

Semi-quantitative analysis was performed using ImageJ (version 1.8) according to a specific protocol for cytoplasmic staining. JPEG images were uploaded into the software, and the IHC Profiler plugin [28] was used to generate numerical values corresponding to immunostaining categories (high positive, positive, low positive, and negative). These values were recorded in a digital spreadsheet and used to calculate an overall intensity score (I-Score) ((high positive × 4 + positive × 3 + low positive × 2 + negative × 1)/100), which yields a numerical value without an associated unit, ranging from 1 to 4. The mean I-score of each fish was then calculated and used for statistical analyses (*n* = 6 fish/month/per marker).

#### 2.5.3. Quantitative Analysis of PCNA Immunolabelling

The semi-quantitative immunolabelling used for the other markers was not applicable to PCNA, as this marker primarily labels cell nuclei, and the labelled cells are widely scattered. Therefore, a different approach was used: stereologically estimating the relative volume of positively stained nuclei within the liver parenchyma by manual point counting on live images captured by a digital camera (EP50, Olympus, Japan) coupled with an optical microscope (CX21, Olympus, Japan), using a ×40 objective lens, and displayed on a monitor (VS247HR, ASUS, Taipei, Taiwan) via software (EPview, Tokyo, Olympus).

**Figure 1:**
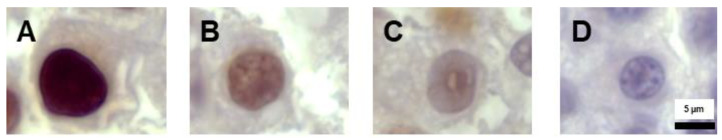
Intensities of hepatocyte nuclear proliferating cell nuclear antigen (PCNA) immunostaining. (**A**–**C**) represent positively stained nuclei, while (**D**) shows an unstained nucleus.

Each sampled liver section was divided into six quadrants. A point-counting grid with 96 points (spaced at 12 µm intervals) was superimposed on two randomly selected fields per quadrant, resulting in 12 fields per section (3 sections/fish; 36 fields/fish). The points overlapping PCNA-positive nuclei were counted and divided by the total number of points hitting the liver, yielding an unbiased classic estimate of the relative volume of the positive nuclei in the liver [29]. All stained nuclei were considered, regardless of immunostaining intensity (Figure 1).

### 2.6. Cell, Nuclear, and Cytoplasmic Volumes, and Nuclear-to-Cell Volume Ratio

The mean hepatocyte volume (${\bar{v}}_{cell}$) and mean nuclear volume (${\bar{v}}_{nucleus}$) were estimated by the single-section nucleator technique as described for fish liver [30]. Briefly, the procedure was implemented semi-automatically using the CAST-Grid System version 1.5 (Olympus Danmark A/S, Søborg, Denmark) and applied to systematically uniform, randomly sampled areas. A sampling grid with forbidden lines was overlaid on the live image. Hepatocytes were included when the nucleolus was focused and located within the sampling frame or touched an inclusion line, but not an exclusion line. The centre of an in-focus nucleolus was used as the unique reference point, from which four isotropic intercepts were drawn to the nuclear or cellular boundary. The ${\bar{v}}_{cell}$ and ${\bar{v}}_{nucleus}$ were then automatically estimated by the software using the nucleator formula: ${\bar{v}}_{particle}$ = (4π) ÷ 3 × *l^3^*, where *l* is the mean intercept length. The cytoplasmic volume (${\bar{v}}_{cytoplasm}$) was computed by subtracting the ${\bar{v}}_{nucleus}$ from the ${\bar{v}}_{cell}$. The nuclear-to-cell volume (N/C) ratio was calculated by dividing the ${\bar{v}}_{nucleus}$ by the ${\bar{v}}_{cell}$. Between 100 and 200 hepatocytes per fish were measured, meeting the recommended sample size to offer a low coefficient of error of the estimates [30,31]. Volumes were expressed in µm^3^, while the N/C ratio was presented as a percentage.

### 2.7. Gene Expression Analysis

#### 2.7.1. RNA Extraction and cDNA Synthesis

One random liver fragment from each fish was extracted using the illustra^TM^ RNAspin Mini Isolation Kit (GE Healthcare, Chicago, IL, USA), which included an in-column DNase I digestion step. RNA quantification was performed using a Multiskan™ GO microplate spectrophotometer (Thermo Fisher Scientific, Vantaa, Finland), with SkanIt software version 4.1 (Thermo Fisher Scientific, Waltham, MA, USA). RNA integrity was assessed by measuring the optical density ratios (260/280 nm and 260/230 nm), together with electrophoresis on a 1% agarose gel stained with GelRed™ (Biotium, Fremont, CA, USA). cDNA was synthesised from 1 µg of total RNA using the iScript™ cDNA Synthesis Kit (Bio-Rad, Hercules, CA, USA) in a final reaction volume of 20 µL.

#### 2.7.2. Quantitative Real-Time Polymerase Chain Reaction (qRT-PCR)

Real-time polymerase chain reaction (qRT-PCR) was performed using a real-time PCR detection system (CFX Connect, Bio-Rad, Hercules, CA, USA). Each reaction had a final volume of 20 µL, consisting of 5 µL of diluted cDNA sample, 10 µL of IQ SYBR Green (Bio-Rad), and 0.4 µL (200 nM) of each primer, plus 4.2 µL of nuclease-free water. Samples were run in duplicate, and no-template controls (NTC) were included. Primer sequences, annealing temperatures, and amplification efficiencies are provided in Table 1. A melting curve analysis was done from 55 °C to 95 °C, increasing the temperature by 0.5 °C every 30 s, to verify the specificity of the amplification products. Relative gene expression was quantified using the Pfaffl method [32]. Expression levels were normalised using the geometric mean of two reference genes, *ribosomal protein L8* (*rpl8*) and *beta actin* (*β-actin*), based on their stability in previous studies [13].

### 2.8. Statistical Analyses

Data were grouped and analysed by sampling month and sex. All analyses were executed using jamovi software (version 2.6.26). Before further analysis, data were tested for normality (Shapiro–Wilk test) and homogeneity of variances (Levene’s test). When these assumptions were violated, logarithmic (log_10_) or Box–Cox transformations were used to ensure normality and homoscedasticity. Differences between sexes, reproductive stages, and their interaction were assessed using a two-way ANOVA, followed by Tukey’s post hoc test when significant effects were detected (*p* < 0.05).

## 3. Results

### 3.1. Hepatosomatic Index (HSI)

**Figure 2:**
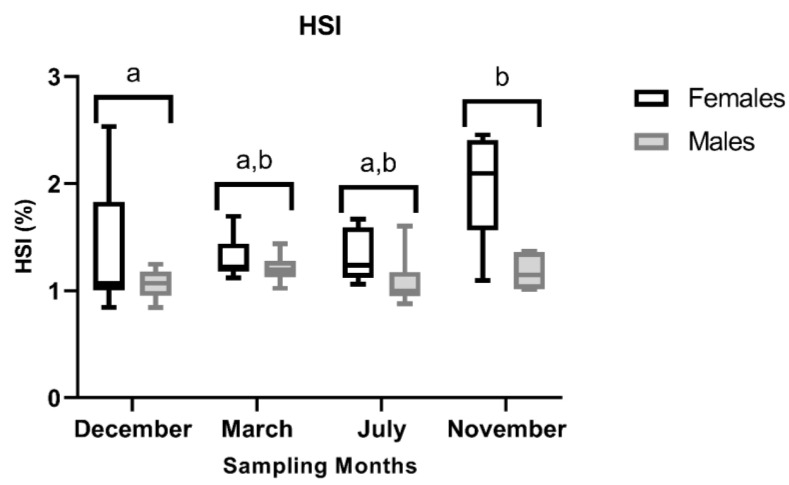
Mean HSI ± standard deviation, calculated as liver-to-body ratio, excluding the gonadal mass, of male and female brown trout (*Salmo trutta*) across the distinct reproductive stages. Data are presented as box plots (median, IQR, and min–max range); *n* = 6 per group. Bars not sharing the same letter (a or b) indicate statistically significant differences (*p* < 0.05) according to Tukey’s post hoc test. Precise *p*-values for all significant comparisons are provided in Supplementary Table S1.

HSI data is given in Figure 2. The ANOVA revealed significant main effects of both month and sex on HSI, with no significant month × sex interaction (month: F_3,40_ = 3.09, *p* = 0.038; sex: F_1,40_ = 11.63, *p* = 0.001; month × sex: F_3,40_ = 1.12, *p* = 0.352). Overall, females (1.49 ± 1.13) showed higher HSI values than males (1.13 ± 0.18). Post hoc comparisons for the month indicated that November had a significantly greater HSI than December (Tukey, *p* = 0.037), whereas the other pairwise contrasts were not significant. Descriptively, female HSI peaked in November, while male HSI remained relatively stable across months.

### 3.2. Immunohistochemistry

#### 3.2.1. Vtg Immunostaining

**Figure 3:**
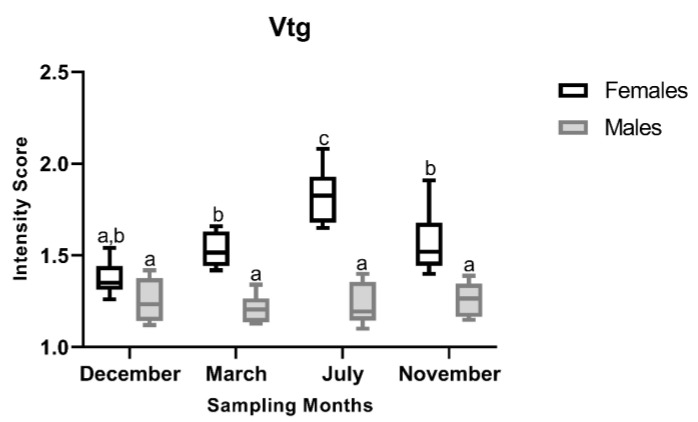
Variation in vitellogenin (Vtg) immunostaining intensity scores in liver sections of male and female brown trout (*Salmo trutta*) across the distinct reproductive stages. Data are presented as box plots (median, IQR, and min–max range); *n* = 6 per group. Bars not sharing the same letter (a, b, or c) indicate statistically significant differences (*p* < 0.05) according to Tukey’s post hoc test. Precise *p*-values for all significant comparisons are provided in Supplementary Table S2.

**Figure 4:**
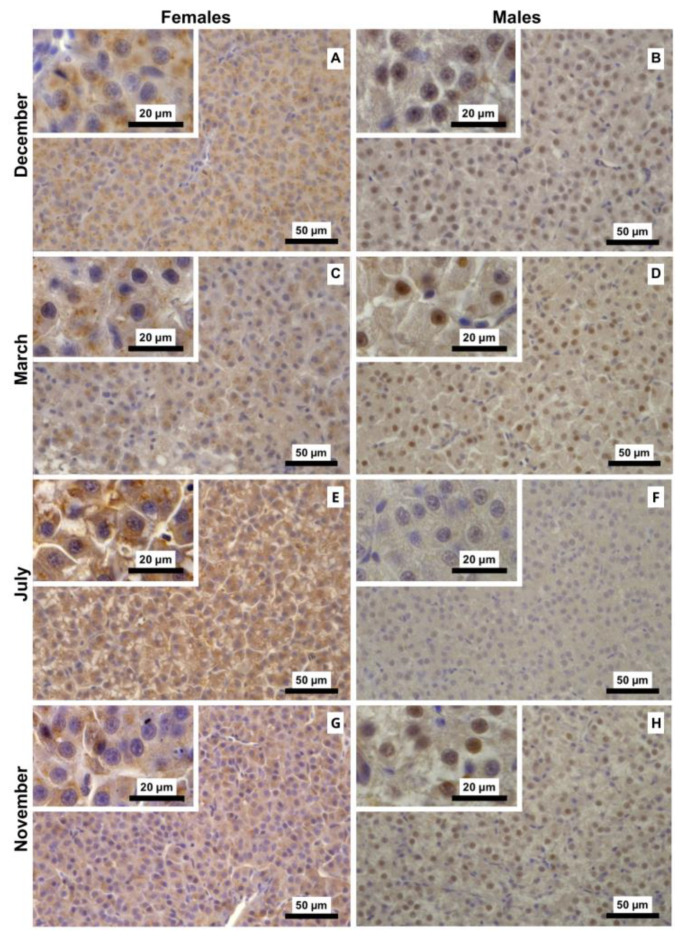
Representative vitellogenin (Vtg) immunostaining of liver sections of male and female brown trout (*Salmo trutta*) across the distinct reproductive stages. (**A**)—females December; (**B**)—males December; (**C**)—females March; (**D**)—males March; (**E**)—females July; (**F**)—males July; (**G**)—females November; and (**H**)—males November. Females consistently exhibit darker brown cytoplasmic staining, evidently stronger in July. In males, cytoplasmic immunostaining is fainter, and nuclei often show positive brown staining.

ANOVA of the Vtg I-score data showed significant main effects for month (F_3,40_ = 3.25, *p* < 0.032), sex (F_1,40_ = 89.20, *p* < 0.001), and the interaction between both factors (F_3,40_ = 4.37, *p* = 0.009), as illustrated in Figure 3. Overall, females had higher I-scores than males across all reproductive stages, a pattern also qualitatively evident in Vtg immunostaining of liver sections, where females displayed a stronger signal (Figure 4). In females, I-score peaked in the regenerating stage (July) and was lowest in the spawning-capable stage (December), with intermediate scores observed in the regressing (March) and developing (November) stages. Tukey’s post hoc test showed that the developing stage (July) I-score was significantly higher than those of all other stages. In contrast, male Vtg I-scores remained consistently low across the distinct stages, with no significant differences (Figure 3). In males, Vtg immunostaining was not strictly cytoplasmic, with hepatocyte nuclei also exhibiting immunoreactivity (Figure 4).

#### 3.2.2. ZP Immunostaining

**Figure 5:**
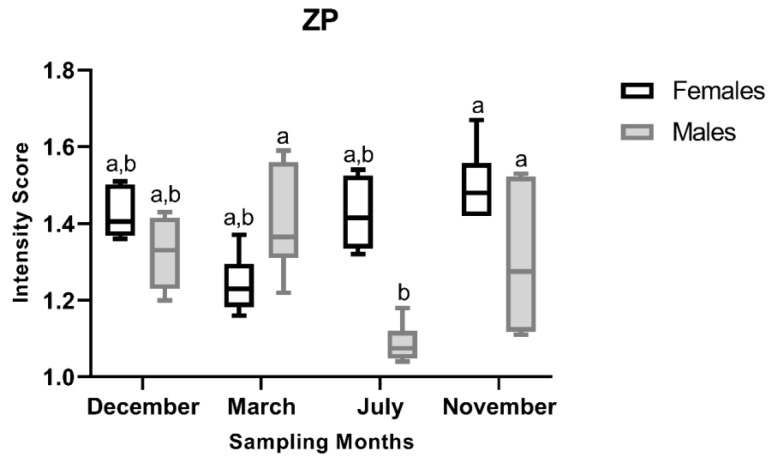
Variation in zona pellucida (ZP) protein immunostaining scores in liver sections of male and female brown trout (*Salmo trutta*) across the distinct reproductive stages. Data are presented as box plots (median, IQR, and min–max range); *n* = 6 per group. Bars not sharing the same letter (a or b) indicate statistically significant differences (*p* < 0.05) according to Tukey’s post hoc test. Precise *p*-values for all significant comparisons are provided in Supplementary Table S3.

**Figure 6:**
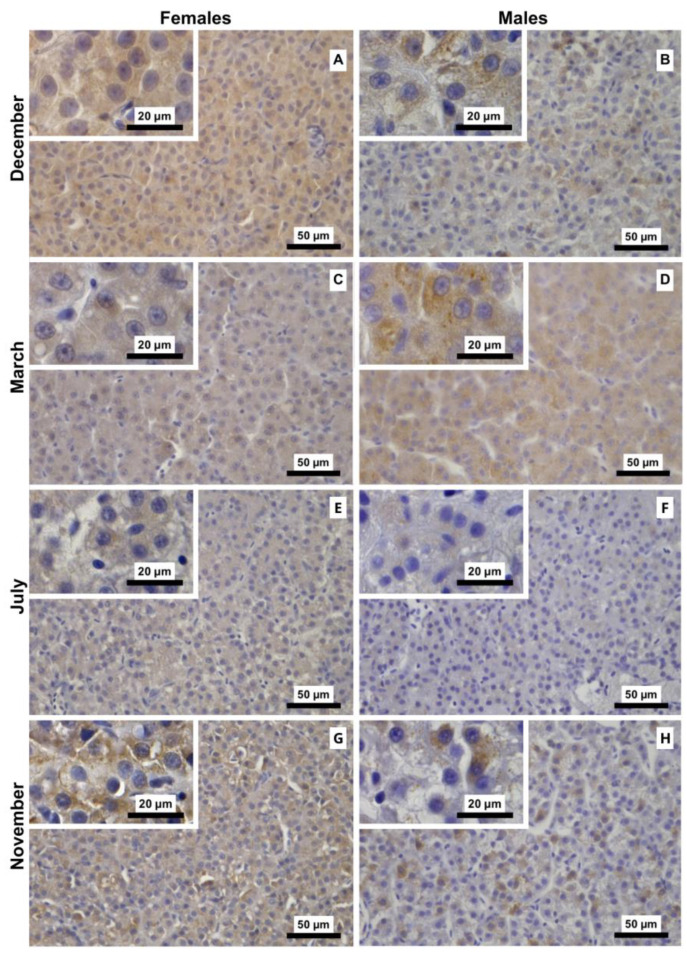
Representative zona pellucida (ZP) protein immunostaining of liver sections from male and female brown trout (*Salmo trutta*) across the distinct reproductive stages. (**A**)—females December; (**B**)—males December; (**C**)—females March; (**D**)—males March; (**E**)—females July; (**F**)—males July; (**G**)—females November; and (**H**)—males November. The average female in December, July, and November tends to show greater intensity than the male counterpart, whereas the July males undoubtedly show less immunostaining. In March, males exhibited a stronger staining pattern compared to females.

ANOVA revealed a significant effect of month (F_3,40_ = 3.94, *p* = 0.014) on ZP I-score, while no significant main effect of sex was detected (F_1,40_ = 0.04, *p* = 0.846), indicating a seasonal pattern in ZP expression. A significant interaction between month and sex was also observed (F_3,40_ = 5.04, *p* = 0.004), indicating a sex-specific pattern, as shown in Figure 5. Female I-scores remained stable across all reproductive stages. In contrast, the male ZP I-scores were the highest during the regressing stage (March), followed by the developing (November) and spawning-capable (December) stages. The lowest I-scores in males were observed in the regenerating stage (July), with Tukey’s post hoc analysis indicating significantly lower intensity in July compared to March and November. This seasonal pattern is also qualitatively evident in images of ZP immunostaining (Figure 6).

#### 3.2.3. Casp3 Immunostaining

**Figure 7:**
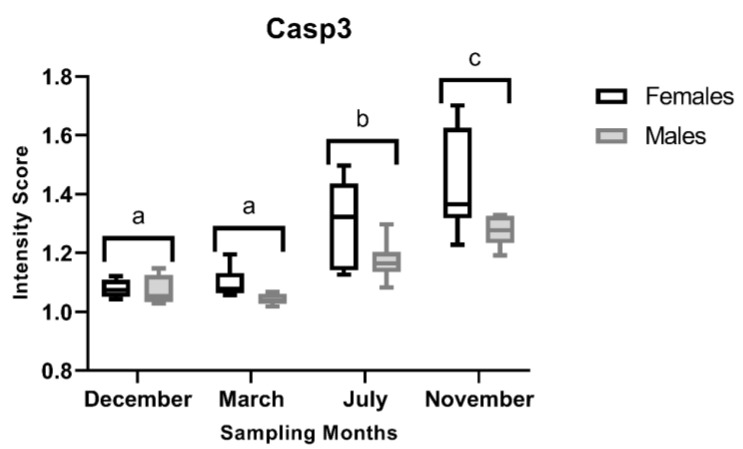
Variation in caspase-3 (Casp3) immunostaining scores in liver sections of male and female brown trout across (*Salmo trutta*) the distinct reproductive stages. Data are presented as box plots (median, IQR, and min–max range); *n* = 6 per group. Bars not sharing the same letter (a, b or c) indicate statistically significant differences (*p* < 0.05) according to Tukey’s post hoc test. Precise *p*-values for all significant comparisons are provided in Supplementary Table S4.

**Figure 8:**
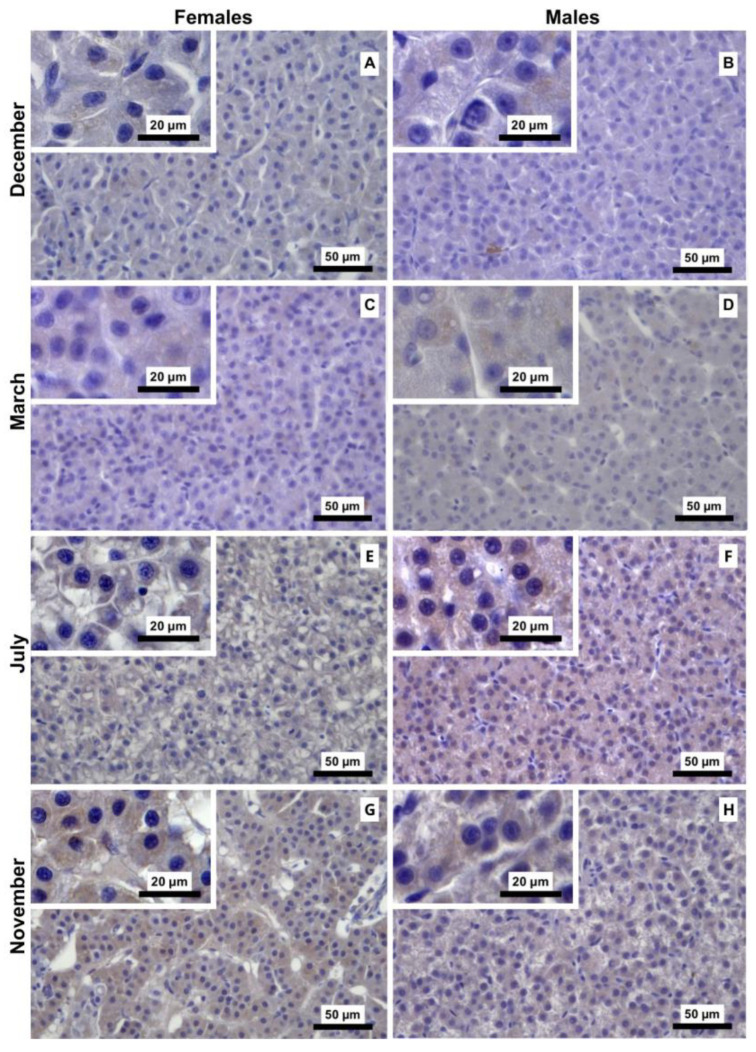
Representative caspase-3 (Casp3) immunostaining of liver sections from male and female brown trout (*Salmo trutta*) across the distinct reproductive stages. (**A**)—females December; (**B**)—males December; (**C**)—females March; (**D**)—males March; (**E**)—females July; (**F**)—males July; (**G**)—females November; and (**H**)—males November. The hepatocytic cytoplasmic staining intensity tends to be similar in animals from December and March, whereas in July and, particularly, in November, the brown tinge appears more intense.

ANOVA revealed significant main effects of month (F_3,40_ = 44.92, *p* < 0.001) and sex (F_1,40_ = 12.22, *p* = 0.001), indicating seasonal variation and sex differences. In contrast, the month × sex interaction was not significant (F_3,40_ = 1.07, *p* = 0.374), suggesting that the sex-related difference did not vary seasonally (Figure 7). Overall, females tended to have higher Casp3 I-scores than males, with the largest sex differences evident in July and November. For both males and females, the highest I-scores were observed in the developing stage (November), followed by the regenerating stage (July), which differed significantly from the regressing (March) and spawning-capable (December) stages. Representative images of Casp3-immunostained liver sections are shown in Figure 8, revealing a pattern consistent with the I-score analysis.

#### 3.2.4. PCNA Immunostaining

**Figure 9:**
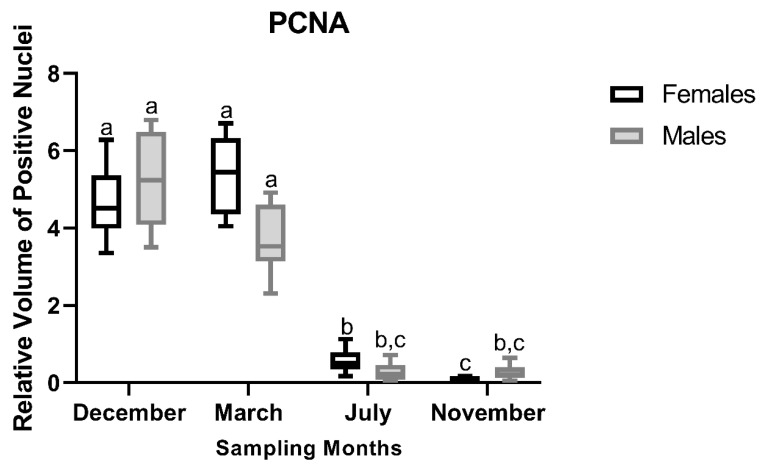
Relative volume (%) of hepatocyte nuclei positive for immunostaining against proliferating cell nuclear antigen (PCNA) in liver sections of male and female brown trout (*Salmo trutta*) across distinct reproductive stages. Data are presented as box plots (median, IQR, and min–max range); *n* = 6 per group. Bars not sharing the same letter (a, b, or c) indicate statistically significant differences (*p* < 0.05) according to Tukey’s post hoc test. Precise *p*-values for all significant comparisons are provided in Supplementary Table S5.

**Figure 10:**
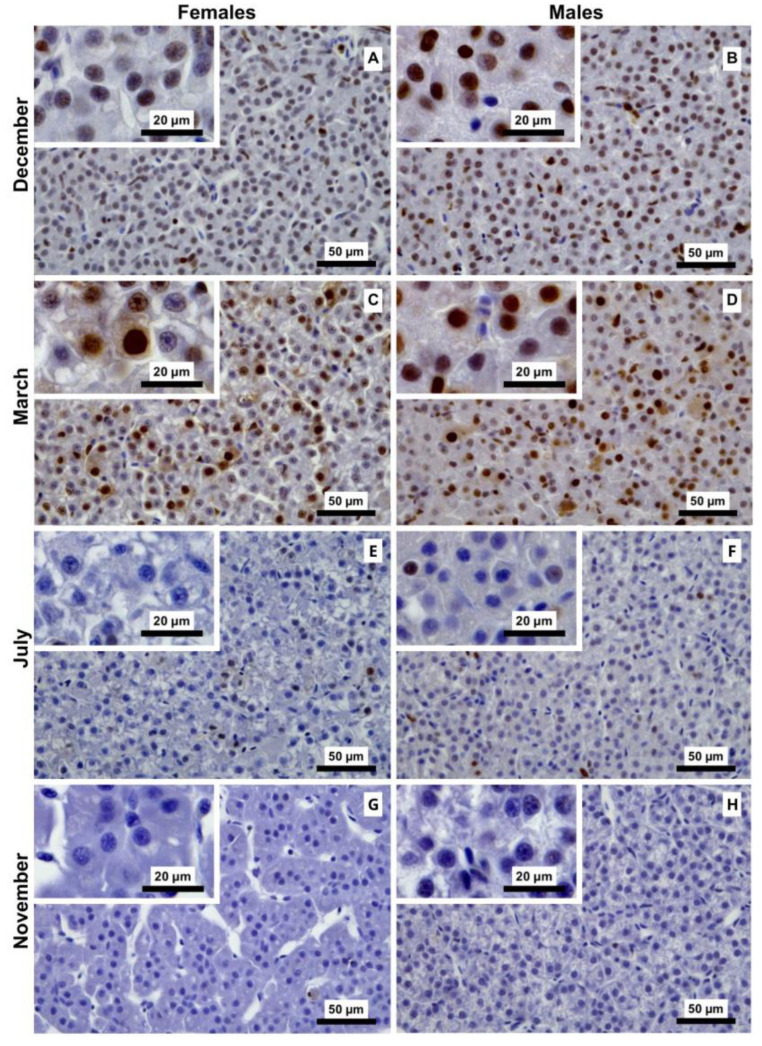
Representative proliferating cell nuclear antigen (PCNA) immunostaining of liver sections from male and female brown trout (*Salmo trutta*) across the distinct reproductive stages. (**A**)—females December; (**B**)—males December; (**C**)—females March; (**D**)—males March; (**E**)—females July; (**F**)—males July; (**G**)—females November; and (**H**)—males November. The images reveal a higher density of immunotagged nuclei in both sexes in December and March than in July and November. In March, although staining appears less pronounced in males, individual variability results in overlaps between both sexes.

ANOVA revealed a significant main effect of month on the abundance of PCNA-stained nuclei (F_3,40_ = 224.18, *p* < 0.001). No significant main effect of sex was observed (F_1,40_ = 1.88, *p* = 0.178); however, a significant month and sex interaction was detected (F_3,40_ = 4.77, *p* = 0.006) (Figure 9). In females, the percentage of PCNA-positive nuclei was highest in the spawning-capable (December) and regressing (March) stages and declined significantly in the remaining months, with Tukey’s post hoc analysis indicating a significant decrease from the regenerating stage (July) to the developing stage (November). In males, PCNA staining followed a similar seasonal pattern, with significantly higher percentages observed in the spawning-capable (December) and regressing (March) stages compared with the regenerating (July) and developing (November) stages. Representative PCNA-immunostained liver sections are shown in Figure 10, illustrating a pattern that is overall compatible with the stereological relative volume estimates. In March, staining tended to be less pronounced in males, but both sexes overlapped.

### 3.3. Cell-Related Volumes and Nuclear-to-Cell Volume Ratio

**Figure 11:**
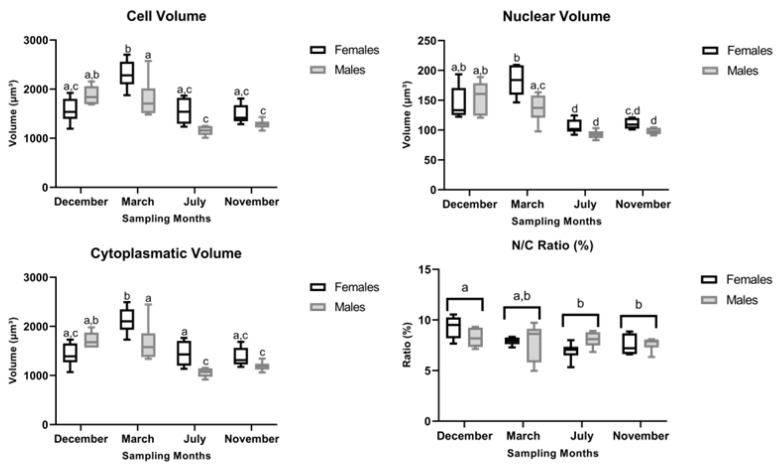
Hepatocyte ${\bar{v}}_{cell}$ (μm^3^), ${\bar{v}}_{nucleus}$(μm^3^), ${\bar{v}}_{cytoplasm}$ (μm^3^) and N/C (nuclear to cell volume) ratio (%) in male and female brown trout (*Salmo trutta*) liver across the reproductive stages. Data are presented as box plots (median, IQR, and min–max range); *n* = 6 per group. Bars not sharing the same letter (a, b, c or d) indicate statistically significant differences (*p* < 0.05) according to Tukey’s post hoc test. Precise *p*-values for all significant comparisons are provided in Supplementary Tables S6–S9.

As to the ${\bar{v}}_{cell}$, ANOVA showed significant main effects of month (F_3,40_ = 22.13, *p* < 0.001), sex (F_1,40_ = 8.10, *p* = 0.007) and their interaction (F_3,40_ = 6.59, *p* = 0.001). Overall, females’ ${\bar{v}}_{cell}$ was higher than that of males, with Tukey’s post hoc test detecting a significant difference between sexes in the regressing stage (March) (Figure 11). The ${\bar{v}}_{cell}$ of females was significantly higher in the regressing stage (March) than in the spawning-capable (December), regenerating (July), and developing (November) stages (Figure 11). As to the ${\bar{v}}_{cell}$ of males, two distinct group profiles were observed: the spawning-capable (December) and regressing (March) stages, versus the regenerating (July) and developing (November) stages (Figure 11). Significant differences were found between the groups, but not within each month (Figure 11).

As to the ${\bar{v}}_{nucleus}$, ANOVA evidenced significant main effects of month (F_3,40_ = 41.06, *p* < 0.001), sex (F_1,40_ = 11.57, *p* = 0.002) and their interaction (F_3,40_ = 2.91, *p* = 0.046). Females’ ${\bar{v}}_{cell}$ was significantly higher than that of males, with Tukey’s post hoc pairwise analysis revealing a significant difference in the regressing stage (March) (Figure 11). The ${\bar{v}}_{nucleus}$ in females peaked in the spawning-capable (December) and regressing (March) stages, differing significantly from the regenerating (July) and developing (November) stages (Figure 11). In males, the ${\bar{v}}_{nucleus}$ followed a similar seasonal pattern, with the spawning-capable (December) and regressing (March) stages showing higher nuclear volumes than the regenerating (July) and developing (November) stages (Figure 11).

The ANOVA results for the ${\bar{v}}_{cytoplasm}$ showed a significant main effect of month (F_3,40_ = 21.37, *p* < 0.001), sex (F_1,40_ = 9.20, *p* = 0.005) and their interaction (F_3,40_ = 7.40, *p* < 0.001). Overall, the females’ ${\bar{v}}_{cytoplasm}$ was higher than that of males. Tukey’s post hoc analysis revealed that this sex-related difference was significant in the regressing (March) and regenerating (July) stages. The seasonal pattern of the ${\bar{v}}_{cytoplasm}$ was similar to the one described for ${\bar{v}}_{cell}$.

Regarding the N/C ratio (Figure 11), the ANOVA reported a significant main effect of month (F_3,40_ = 3.83, *p* = 0.017) and no significant effect of sex (F_1,40_ = 0.01, *p* = 0.914) or the interaction between these variables (F_3,40_ = 2.34, *p* = 0.088). Overall, the N/C ratio was higher in the spawning-capable stage (December), followed by the regressing stage (March), the developing stage (November), and the regenerating stage (July).

### 3.4. qRT-PCR

#### 3.4.1. VtgA Expression

**Figure 12:**
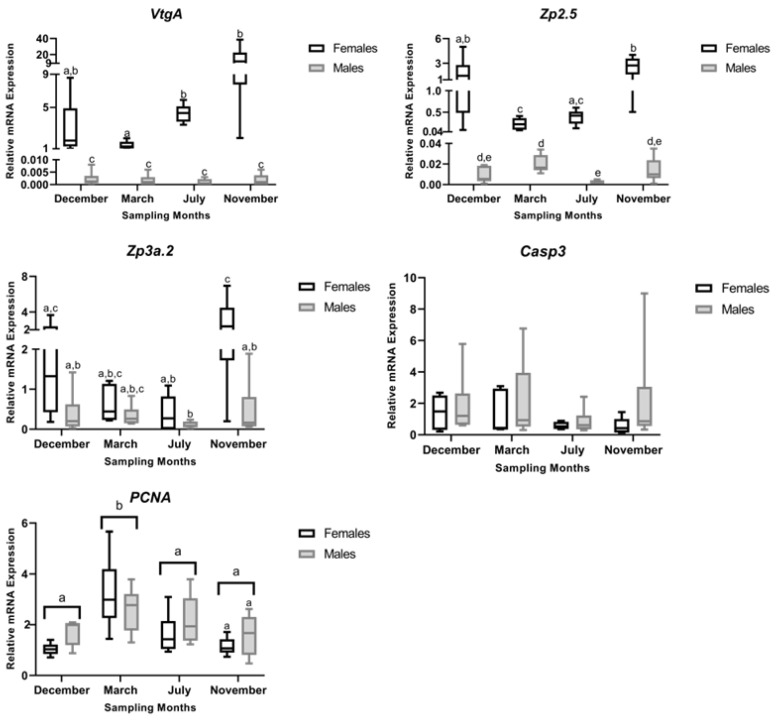
Relative liver mRNA expression of *vitellogenin A* (*VtgA*); *zona pellucida glycoprotein 2.5* (*Zp2.5*); *zona pellucida glycoprotein 3a.2* (*Zp3a.2*); *caspase-3* (*Casp3*); and *proliferating cell nuclear antigen* (*PCNA*) in male and female brown trout (*Salmo trutta*) across the sampling months. Data are presented as box plots (median, IQR, and min-max range); *n* = 6 per group. Bars not sharing a letter (a, b, c, d or e) indicate significant differences (*p* < 0.05) according to the Tukey test. Precise *p*-values for all significant comparisons are provided in Supplementary Tables S10–S13.

For *VtgA* mRNA levels, significant main effects of month (F_3,40_ = 3.20, *p* = 0.033), sex (F_1,40_ = 321.96, *p* < 0.001), and their interaction (F_3,40_ = 5.61, *p* = 0.003) were found (Figure 12). Tukey’s post hoc test indicated that females had significantly higher *VtgA* mRNA expression than males in all reproductive stages. In females, expression levels were highest in the developing stage (November), followed by the regenerating (July) and spawning-capable (December) stages. In contrast, the lowest expression was observed in the regressing stage (March), which differed significantly from the developing (November) and regenerating (July) stages (Figure 12). Conversely, *VtgA* mRNA levels in males remained consistently low across all stages.

#### 3.4.2. Zp2.5 Expression

The ANOVA of *Zp2.5* mRNA levels showed significant effects of month (F_3,40_ = 6.97, *p* < 0.001), sex (F_1,40_ = 255.15, *p* < 0.001) and their interaction (F_3,40_ = 7.91, *p* < 0.001). Expression was significantly higher in females than in males (Figure 12). In females, *Zp2.5* mRNA expression levels were highest at the developing stage (November), followed by the spawning-capable stage (December), with no significant difference between these stages. Expression levels in the regenerating (July) and regressing (March) stages did not differ from each other, but were significantly lower than those in the developing (November) and spawning-capable (December) stages (Figure 12). In males, *Zp2.5* expression remained at basal levels, with minimal relative expression detected. Nevertheless, Tukey’s post hoc test revealed a significant difference between the regressing stage (March) and the regenerating stage (July). In contrast, expression levels at the developing (November) and spawning-capable (December) stages did not differ significantly from each other or from those observed in the other reproductive stages (Figure 12).

#### 3.4.3. Zp3a.2 Expression

The ANOVA revealed significant main effects of month (F_3,40_ = 5.18, *p* = 0.004) and sex (F_1,40_ = 11.40, *p* = 0.002), but no significant interaction between month and sex (F_3,40_ = 1.74, *p* = 0.173) (Figure 12). Post hoc comparisons indicated that expression levels were highest at the developing stage (November) and lowest in the regenerating stage (July), with the developing (December) and regressing (March) stages showing intermediate values. Across months, females exhibited higher expression than males. The absence of a significant interaction indicates that the seasonal pattern was comparable between sexes (Figure 12).

#### 3.4.4. Casp3 Expression

For *Casp3*, no significant effects of month (F_3,40_ = 1.281, *p* = 0.294), sex (F_1,40_ = 2.997, *p* = 0.091), or their interaction (F_3,40_ = 0.413, *p* = 0.745) were found, revealing stable *Casp3* mRNA expression across all reproductive stages in both sexes (Figure 12).

#### 3.4.5. PCNA Expression

The ANOVA revealed a significant main effect of month (F_3,40_ = 9.24, *p* < 0.001), whereas neither sex (F_1,40_ = 1.24, *p* = 0.272) nor the month × sex interaction had a significant effect (F_3,40_ = 1.59, *p* = 0.207). The regressing stage (March) showed the greatest expression, with mRNA levels significantly higher than in all other stages.

## 4. Discussion

Despite the central role of the liver in reproductive regulation, most studies on the reproductive cycle have focused almost exclusively on gonadal tissues [8,9,10], leaving hepatic regulation largely uncharacterised. Furthermore, studies directly comparing male and female individuals across the reproductive cycle remain scarce. The reproductive stages examined in the present study were previously characterised based on gonadal analyses of the same animals [10]. The current investigation extends existing knowledge by providing novel insights into the hepatic protein and gene expression dynamics of Vtg and ZP, two key reproductive regulators. By integrating markers of cell proliferation (PCNA) and programmed cell death (Casp3), our results further reveal sex-specific and seasonal patterns of liver functional remodelling. Our data showed inter-individual variation at each reproductive stage, likely reflecting that, within a given phase, fish were not fully synchronised in gonadal development or plasma endocrine status, as previously reported [10].

### 4.1. Hepatic Reproductive Regulators

Our findings support the classical view that oestrogen activity regulates Vtg and ZP synthesis, as females showed upregulation of the corresponding genes during the developing stage (November), concurrent with peak plasma E2 levels [10]. Elevated *ZP* mRNA levels persisted through the spawning-capable stage (December), indicating sustained regulation beyond the initial oestrogen peak, followed by an eventual decrease after spawning. The relationship between gene expression and cellular protein abundance is not necessarily linear [36]. Consistent with this, our data reveal discrepancies between transcript levels and protein detection. In females, Vtg showed increased immunosignalling in July, indicating greater protein retention at the regenerating stage compared with other reproductive stages, whereas ZP protein signalling remained relatively stable across the distinct reproductive stages. This pattern contrasts with transcript dynamics, as higher protein immunostaining would plausibly be expected during stages with higher gene expression. Such decoupling may reflect increased mobilisation of essential reproductive proteins during the later stages of development, when hepatic reserves are redirected to the gonad to meet increased reproductive demands. Corroborating this view, a previous study by Chen et al. reported that Vtg plasma levels in rainbow trout peak in September and decline in the following months [37]. That study also showed that around July, the hepatosomatic index (HSI) lowers while the gonadosomatic index (GSI) increases, suggesting that hepatic resources are being mobilised from the liver to the gonad. Our previous study reported that brown trout female GSI reached its lowest values in July (regenerating stage) and peaked in November (developing stage) and December (spawning-capable stage), further supporting the mentioned physiological mechanism [10]. The increased Vtg immunosignalling observed during the regenerating stage (July) may be associated with greater hepatic retention during the early stages of vitellogenesis—the accumulation of which could contribute to the peak of HSI we detected here in November—while ZP proteins appear to maintain comparatively stable reserves.

In adult male salmonids, hepatic Vtg and ZP expression is generally considered negligible and is therefore widely used as a biomarker of exposure to estrogenic compounds [11,12,38]. Nevertheless, low-level expression or detection of these proteins has been reported in males of other teleost species [15,16,18]; however, variation across the reproductive cycle has not been characterised. In this context, our data suggest that male brown trout can express both Vtg and ZP. *VtgA* mRNA levels in males, although detected at much lower levels than in females, were observed across all reproductive stages. A consistently low but distinct protein signal was also detected, suggesting constitutive baseline expression likely independent of reproductive activity and potentially linked to the low endogenous E2 levels measured in these specimens [10].

Nevertheless, the detection of nuclear Vtg immunolabelling in male hepatocytes was unexpected, as Vtg is classically characterised as a secreted yolk precursor protein with no known nuclear or DNA-binding function. A direct role as a transcription factor, therefore, appears highly improbable. Notably, nuclear staining was consistently absent in females, despite their markedly higher physiological vitellogenin production, making a simple, non-specific staining artefact unlikely. The sex-restricted nuclear signal instead supports a biological explanation, most plausibly related to sex-dependent differences in intracellular processing, trafficking, or accumulation of vitellogenin-derived fragments recognised by the antibody. While some degree of antibody cross-reactivity with nuclear components cannot be categorically excluded, it does not adequately explain the reproducible and sex-specific localisation pattern observed here. The significant fluctuation in ZP expression was also surprising, and interestingly, both mRNA levels and immunostaining patterns coincided, suggesting a decrease at the regenerating stage (July), a pattern not readily explained by endocrine activity. Although this detection can theoretically be induced by xenoestrogens, this is highly unlikely in our study, as the aquaculture is located deep in the mountains and is fed by the pristine Marão River. Additionally, the data from Santos et al. showed normal E2 and T levels and typical gonadal maturation patterns, providing no evidence of xenoestrogenic contamination [10]. Together, these findings support that intrinsic differences in basal Vtg and ZP expression exist among salmonid males and demonstrate that at least adult male brown trout can express and produce these proteins.

### 4.2. Liver Seasonal Remodelling

In salmonids, the liver is well established as highly plastic, undergoing structural remodelling throughout the breeding cycle. Previously, it has been reported that the HSI in brown trout tends to rise leading up to and during spawning, and to decline following the completion of reproduction [10,20]. Furthermore, there is evidence of a positive link between sex steroids and hepatocyte proliferative activity [21]. Our study explored this hypothesis by incorporating markers of proliferation and apoptosis, although changes in HSI cannot be exclusively attributed to proliferative activity across the reproductive stages.

PCNA is expressed in cells actively engaged in DNA replication, being predominantly detected during late G1 and S phases, shortly before mitosis [39]. Consequently, PCNA protein detection by immunohistochemistry reflects a narrow temporal window of the cell cycle. In contrast, mRNA expression reflects a transcriptional activation that may be maintained over longer time periods, allowing detection beyond the brief window in which PCNA protein is immunohistochemically detectable [36].

Seasonal changes in liver size have previously been attributed primarily to fluctuations in hepatocyte number, with increases observed from endogenous to exogenous vitellogenesis [20]. In agreement with these observations, although not statistically significant, we observed an increase in female HSI from the regressing stage (March) to the developing stage (November), followed by a decrease during the spawning-capable stage (December) [10]. Consistent with this pattern, *PCNA* mRNA levels peaked during the regressing stage (March) and remained relatively stable between the regenerating (July) and spawning-capable (December) stages. The increased PCNA immunosignal observed during the spawning-capable stage (December) may appear counterintuitive; however, immunolabelling reflects only the proportion of cells actively cycling at a given moment, rather than the overall proliferative activity of the tissue [40]. Thus, these findings suggest that discrete waves of hepatocyte mitosis may still occur even when global liver proliferation is reduced. Consistently, the increase in hepatocyte size observed during the spawning-capable (December) and regressing (March) stages is compatible with mitotic activity, as cells are known to increase in volume before mitosis [41]. Moreover, despite sex-specific differences in oestrogen-responsive gene expression (e.g., *VtgA* and *Zp2.5*), no corresponding differences were observed in the N/C ratio. This likely reflects proportional increases in both nuclear and cytoplasmic volumes, preserving the ratio. The N/C ratio is a highly conserved cellular feature across eukaryotic cells [42], and is often maintained despite physiological changes in cell activity. Together, these findings indicate that sex-specific transcriptional activation does not necessarily translate into significant changes in global hepatocyte morphology.

In contrast to *PCNA*, *Casp3* transcript levels remained relatively stable across the reproductive cycle, while marked changes were observed at the protein level. This pattern suggests that hepatic apoptosis is not primarily driven by transcriptional modulation of *Casp3*, but rather by changes in apoptotic competence associated with tissue remodelling. The maintenance of stable *Casp3* mRNA expression across stages further supports the notion that apoptotic capacity is constitutively maintained, whereas its engagement is modulated at the protein level in response to physiological demands. In November, the higher Casp3 I-scores likely reflect an increased proportion of hepatocytes undergoing caspase activation during tissue remodelling, without requiring significant changes in steady-state *Casp3* mRNA levels. Taken together, the PCNA and Casp3 patterns indicate that liver remodelling is not uniform across the reproductive cycle. Apoptosis appears more pronounced during the regenerating (July) and developing (November) stages, when liver structure is being remodelled. In contrast, in the spawning-capable (December) and regressing (March) stages, proliferative activity is higher, consistent with the need to repopulate or maintain hepatic tissue after periods of intense mobilisation of hepatic resources (e.g., vitellogenic proteins and energy reserves) towards the gonads. Finally, we observed no direct evidence linking steroid endocrine activity to liver turnover, suggesting that these processes may be regulated by factors independent of direct steroid signalling.

## 5. Conclusions

We examined liver changes in brown trout at different reproductive cycle stages using molecular, immunohistochemical, and stereological methods. This combined framework allowed us to relate gene expression to protein dynamics and better understand how liver function and structure adapt across distinct reproductive stages. Our results highlight sex differences and bring much-needed insight into male profiles, which remain poorly described in the literature. Together, these results show that liver remodelling is strongly sex-specific and stage-dependent, with apoptosis and proliferation peaking at different times and with partial decoupling between transcript levels, protein abundance, and hepatocyte size. Beyond mapping seasonal dynamics and identifying what remains constant, our data provide a reference point for future studies in fish physiology, aquaculture, and environmental monitoring, while paving the way for mechanistic studies on hormone and ecological modulation of liver remodelling in fish.

## Figures and Tables

**Table 1:** Primer sequences, annealing temperatures (AT), and respective efficiencies (E) used for quantitative real-time polymerase chain reaction (qRT-PCR).

Gene	Primer Sequence (5′-3′)	AT (°C)	E (%)	Reference
*Vitellogenin A* (*VtgA*)	F—AACGGTGCTGAATGTCCATAG R—ATTGAGATCCTTGCTCTTGGTC	62.9	99.0	[33]
*Zona pellucida glycoprotein* (*ZP2.5*)	F—ATCAATAACCACAGCCACAATG R—ACCAGGGACAGCCAATATG	55.0	99.0	[21]
*Zona pellucida glycoprotein 3a.2* (*ZP3a.2*)	F—AACTACACTCCACTTCATC R—CACATCTCCTTCATCTTCA	54.5	101.8	[21]
*Caspase 3* (*Casp3*)	F—ACAGCAAAGAGCTAGAGGTCCAACAC R—AAAGCCAGGAGACTTTGACGCAG	56	94.3	[34]
*Proliferating Cell Nuclear Antigen* (*PCNA*)	F—CAGGGATCCATCCTGAAGAA R—GTCCTCATTCCCAGCACACT	61	109.5	[35]
*Beta actin* (*β-act*)	F—TCTGGCATCACACCTTCTAC R—TTCTCCCTGTTGGCTTTGG	55.0	96.1	[14]
*Ribosomal protein L8* (*rpl8*)	F—TCAGCTGAGCTTTCTTGCCAC R—AGGACTGAGCTGTTCATTGCG	59	93.8	[33]

## Data Availability

The original contributions presented in this study are included in the article/Supplementary Material. Further inquiries can be directed to the corresponding author(s).

## Supplementary Materials

The following supporting information can be downloaded at: https://www.mdpi.com/article/10.3390/ani16071073/s1. Table S1. Significant pairwise comparisons of hepatosomatic index (HSI) across months based on Tukey’s post hoc test, Table S2. Significant pairwise comparisons of vitellogenin immunostaining intensity scores across months and sexes based on Tukey’s post hoc test, Table S3. Significant pairwise comparisons of zona pellucida (ZP) protein immunostaining intensity scores across months and sexes based on Tukey’s post hoc test, Table S4. Significant pairwise comparisons of caspase-3 (Casp3) immunostaining intensity scores across months based on Tukey’s post hoc test, Table S5. Significant pairwise comparisons of the relative volume (%) of proliferating cell nuclear antigen (PCNA)-positive hepatocyte nuclei across months and sexes based on Tukey’s post hoc test, Table S6. Significant pairwise comparisons of the hepatocyte cell volume—${\bar{v}}_{cell}$ (μm^3^)—across months and sexes based on Tukey’s post hoc test, Table S7. Significant pairwise comparisons of the hepatocyte nuclear volume—${\bar{v}}_{nucleus}$ (μm^3^)—across months and sexes based on Tukey’s post hoc test, Table S8. Significant pairwise comparisons of the hepatocyte cytoplasmic volume—${\bar{v}}_{cytoplasm}$ (μm^3^)—across months and sexes based on Tukey’s post hoc test, Table S9. Significant pairwise comparisons of the hepatocyte nuclear-to-cell volume ratio —N/C Ratio (%) —across months based on Tukey’s post hoc test. Table S10. Significant pairwise comparisons of the relative liver mRNA expression of *vitellogenin A* (*VtgA*) across months and sexes based on Tukey’s post hoc test, Table S11. Significant pairwise comparisons of the relative liver mRNA expression of *zona pellucida glycoprotein 2.5* (*Zp2.5*) across months and sexes based on Tukey’s post hoc test, Table S12. Significant pairwise comparisons of the relative liver mRNA expression of *zona pellucida glycoprotein 3a.2* (*Zp3a.2*) across months and sexes based on Tukey’s post hoc test, Table S13. Significant pairwise comparisons of the relative liver mRNA expression of *proliferating cell nuclear antigen (PCNA*) across months based on Tukey’s post hoc test.
