# Supplementary material for: Seasonal and Sex-Specific Liver Plasticity in Brown Trout: Estrogen-Responsive Targets and Cell Turnover Dynamics
Source: Animals (Basel). 2026 Apr 1;16(7):1073. doi: 10.3390/ani16071073 (PMC13072334; doi:10.3390/ani16071073)
Supplement: Supplementary file 1 [file animals-16-01073-s001.zip › animals-4207641-supplementary.pdf]

Table S1. Significant pairwise comparisons of hepatosomatic index (HSI) in brown trout (*Salmo trutta*) across months based on Tukey's post-hoc test.

Parameter	Comparison Group A	Comparison Group B	*p* value
**HSI**	December	November	0.037

Table S2. Significant pairwise comparisons of vitellogenin immunostaining intensity scores in brown trout (*Salmo trutta*) across months and sexes based on Tukey's post-hoc test.

Parameter	Comparison Group A	Comparison Group B	*p* value
**Vitellogenin Immunostaining**	December Female	July Female	< 0.001
	December Male	March Female	0.003
	December Male	July Female	< 0.001
	December Male	November Female	< 0.001
	March Female	March Male	< 0.001
	March Female	July Female	0.016
	March Female	July Male	0.001
	March Female	November Male	0.005
	March Male	July Female	< 0.001
	March Male	November Female	< 0.001
	July Female	July Male	< 0.001
	July Female	November Female	0.049
	July Female	November Male	< 0.001
	July Male	November Female	< 0.001
	November Female	November Male	0.001

Table S3. Significant pairwise comparisons of zona pellucida proteins (ZP) immunostaining intensity scores in brown trout (*Salmo trutta*) across months and sexes based on Tukey's post-hoc test.

Parameter	Comparison Group A	Comparison Group B	*p* value
**ZP Immunostaining**	March Male	July Male	0.003
	July Male	November Female	0.002
	July Male	November Male	0.005

Table S4. Significant pairwise comparisons of caspase-3 (Casp3) immunostaining intensity scores across months based on Tukey's post-hoc test.

Parameter	Comparison Group A	Comparison Group B	*p* value
**Casp3 Immunostaining**	December	July	< 0.001
	December	November	< 0.001
	March	July	< 0.001
	March	November	< 0.001
	July	November	0.025

Table S5. Significant pairwise comparisons of the relative volume (%) of proliferating cell nuclear antigen (PCNA)-positive hepatocyte nuclei in brown trout (*Salmo trutta*) across months and sexes based on Tukey's post-hoc test.

Parameter	Comparison Group A	Comparison Group B	*p* value
**PCNA Immunostaining**	December Female	July Female	< 0.001
	December Female	July Male	< 0.001
	December Female	November Female	< 0.001
	December Female	November Male	< 0.001
	December Male	July Female	< 0.001
	December Male	July Male	< 0.001
	December Male	November Female	< 0.001
	December Male	November Male	< 0.001
	March Female	July Female	< 0.001
	March Female	July Male	< 0.001
	March Female	November Female	< 0.001
	March Female	November Male	< 0.001
	March Male	July Female	< 0.001
	March Male	July Male	< 0.001
	March Male	November Female	< 0.001
	March Male	November Male	< 0.001
	July Female	November Female	0.048

Table S6. Significant pairwise comparisons of the hepatocyte cell volume — $\bar{v}_{cell}$ (μm³) — in brown trout (*Salmo trutta*) across months and sexes based on Tukey's post-hoc test.

Parameter	Comparison Group A	Comparison Group B	p value
$\bar{v}_{cell}$	December Female	March Female	< 0.001
	December Male	July Male	< 0.001
	December Male	November Male	0.003
	March Female	March Male	0.018
	March Female	July Female	< 0.001
	March Female	July Male	< 0.001
	March Female	November Female	< 0.001
	March Female	November Male	< 0.001
	March Male	July Male	< 0.001
	March Male	November Male	0.014

Table S7. Significant pairwise comparisons of the hepatocyte nuclear volume — $\overline{v}_{nucleus}$ (μm³) — in brown trout (*Salmo trutta*) across months and sexes based on Tukey's post-hoc test.

Parameter	Comparison Group A	Comparison Group B	p value
$\overline{v}_{nucleus}$	December Female	July Female	0.003
	December Female	July Male	< 0.001
	December Female	November Female	0.029
	December Female	November Male	< 0.001
	December Male	July Female	< 0.001
	December Male	July Male	< 0.001
	December Male	November Female	0.004
	December Male	November Male	0.003
	March Female	March Male	0.043
	March Female	July Female	< 0.001
	March Female	July Male	< 0.001
	March Female	November Female	< 0.001
	March Female	November Male	< 0.001
	March Male	July Female	0.026
	March Male	July Male	< 0.001
	March Male	November Male	0.014

Table S8. Significant pairwise comparisons of the hepatocyte cytoplasmic volume — $\overline{v}_{cytoplasm}$ (μm³) — in brown trout (*Salmo trutta*) across months and sexes based on Tukey's post-hoc test.

Parameter	Comparison Group A	Comparison Group B	p value
$\overline{\boldsymbol{v}}_{\boldsymbol{cytoplasm}}$	December Female	March Female	< 0.001
	December Male	July Male	< 0.001
	December Male	November Male	0.007
	March Female	March Male	0.030
	March Female	July Female	< 0.001
	March Female	July Male	< 0.001
	March Female	November Female	< 0.001
	March Female	November Male	< 0.001
	March Male	July Male	0.001
	March Male	November Male	0.021
	July Female	July Male	0.016

Table S9. Significant pairwise comparisons of the hepatocyte nuclear-to-cell volume ratio — N/C Ratio (%) —across months based on Tukey's post-hoc test.

Parameter	Comparison Group A	Comparison Group B	p value
**N/C Ratio**	December	July	0.021
	December	November	0.036

Table S10. Significant pairwise comparisons of the relative liver mRNA expression of *vitellogenin A* (*VtgA*) in brown trout (*Salmo trutta*) across months and sexes based on Tukey's post-hoc test.

Parameter	Comparison Group A	Comparison Group B	*P* value
**Expression of *VtgA***	December Female	December Male	< 0.001
	December Female	March Male	< 0.001
	December Female	July Male	< 0.001
	December Female	November Male	< 0.001
	December Male	March Female	< 0.001
	December Male	July Female	< 0.001
	December Male	November Female	< 0.001
	March Female	March Male	< 0.001
	March Female	July Female	0.030
	March Female	July Male	< 0.001
	March Female	November Female	0.002
	March Female	November Male	< 0.001
	March Male	July Female	< 0.001
	March Male	November Female	< 0.001
	July Female	July Male	< 0.001
	July Female	November Male	< 0.001
	July Male	November Female	< 0.001
	November Female	November Male	< 0.001

Table S11. Significant pairwise comparisons of the relative liver mRNA expression of *zona pellucida glycoprotein 2.5* (*Zp2.5*) in brown trout (*Salmo trutta*) across months and sexes based on Tukey's post-hoc test.

**Parameter**	**Comparison Group A**	**Comparison Group B**	***p* value**
**Expression of *Zp2.5***	December Female	December Male	< 0.001
	December Female	March Female	0.036
	December Female	March Male	< 0.001
	December Female	July Male	< 0.001
	December Female	November Male	< 0.001
	December Male	March Female	< 0.001
	December Male	July Female	< 0.001
	December Male	November Female	< 0.001
	March Female	March Male	0.011
	March Female	July Male	< 0.001
	March Female	November Female	0.001
	March Female	November Male	< 0.001
	March Male	July Female	< 0.001
	March Male	July Male	0.003
	March Male	November Female	< 0.001
	July Female	July Male	< 0.001
	July Female	November Female	0.036
	July Female	November Male	< 0.001
	July Male	November Female	< 0.001
	November Female	November Male	< 0.001

Table S12. Significant pairwise comparisons of the relative liver mRNA expression of *zona pellucida glycoprotein 3a.2* (*Zp3a.2*) in brown trout (*Salmo trutta*) across months and sexes based on Tukey's post-hoc test.

**Parameter**	**Comparison Group A**	**Comparison Group B**	***p* value**
**Expression of *Zp3a.2***	December Female	July Male	0.033
	December Male	November Female	0.028
	July Female	November Female	0.005
	July Male	November Female	0.001
	November Female	November Male	0.044

Table S13. Significant pairwise comparisons of the relative liver mRNA expression of *proliferating cell nuclear antigen (PCNA)* in brown trout (*Salmo trutta*) across months based on Tukey's post-hoc test.

**Parameter**	**Comparison Group A**	**Comparison Group B**	***p* value**
**Expression of *PCNA***	December	March	< 0.001
	March	July	0.025
	March	November	< 0.001
